# Role of OCT4 in the Regulation of FSH-Induced Granulosa Cells Growth in Female Mice

**DOI:** 10.3389/fendo.2019.00915

**Published:** 2020-01-10

**Authors:** Dai Heng, Qiaozhi Wang, Xiaoshu Ma, Ye Tian, Kaili Xu, Xuechun Weng, Xusong Hu, Wenbo Liu, Cheng Zhang

**Affiliations:** College of Life Science, Capital Normal University, Beijing, China

**Keywords:** FSH, OCT4, GSK3β, β-catenin, granulosa cells, mice

## Abstract

As a member of the POU (Pit-Oct-Unc) transcription factor family, OCT4 (Octamer-binding transcription factor 4) is associated with the cellular proliferative. However, the roles of OCT4 in regulating the transition from preantral follicle to early antral follicle are still remains unclear. To evaluate the effect of OCT4 on cellular development in ovary, mice were injected with eCG *in vivo* or granulosa cells were co-cultured with FSH *in vitro*. The results showed that eCG up-regulated ovarian OCT4 expression. Meanwhile, OCT4 expression in granulosa cells was also up-regulated by FSH, and knockdown of OCT4 by siRNA significantly decreased FSH-induced cellular viability. Moreover, gonadotropin increased p-GSK3β (Glycogen synthase kinase 3-beta) level, β-catenin expression and its translocation to nuclear in ovarian cells. In addition, the inhibition of GSK3β activity by CT99021 significantly increased the expression of β-catenin and OCT4 in granulosa cells. And knockdown β-catenin by siRNA dramatically abolished FSH-induced OCT4 expression and cellular development. Furthermore, FSH-induced the phosphorylation of GSK3β, expression of β-catenin and OCT4, and translocation of β-catenin were mediated by the PI3K/Akt pathway. Taken together, the present study demonstrates that FSH regulated OCT4 expression via GSK3β/β-catenin pathway, which was mediated by the PI3K/Akt pathway. And these regulations are involved in ovarian cell development.

## Introduction

Mammalian ovarian follicular development is a highly selective process that is complex and accompanied by cell proliferation, differentiation, or apoptosis (1–6). The transition from preantral follicle to early antral follicle is a critical period for follicular development (development vs. atresia), which is responsive to gonadotropin (4). Our previous study show that FSH increases preantral follicle growth *in vitro* (7). Moreover, FSH significantly promotes granulosa cell proliferation, inhibits cellular apoptosis, which is mediated by the phosphatidylinositol 3-kinase (PI3K)/protein kinase B (Akt) pathway (8, 9). However, the precise mechanisms of FSH-induced cellular growth are still unclear.

Octamer-binding transcription factor 4 (OCT4) is known as a stem-cells marker, which is closely related with the various cells development (10–14). OCT4 can promote ovarian mesenchymal cell proliferation and increase the potency to promote oogenesis, and regulates the development of membrane cells and granulosa cells (15). It has been reported that OCT4 is detected in oocyte and granulosa cell of growing follicles, and plays important roles in oocyte growth and meiosis (14, 16, 17). Moreover, the expression of OCT4 in ovary is closely related to the estrous cycle, which is also regulated by gonadotropin (18–20). However, the precise roles and mechanisms of OCT4 during the early follicular development are not known.

It has been reported that β-catenin is very important to the stem cell proliferation and hair follicle regeneration by regulating OCT4 expression (21, 22). The Wnt/β-catenin signaling is involved in the regulations of follicular development (23, 24), which is regulated by GSK3β. GSK3β, as an enzyme, regulates the stabilization of β-catenin and its translocation to the nucleus (25). And GSK3β also regulates cell development, energy metabolism, gene transcription, cell cycle progression, proliferation, and apoptosis *in vivo* (26, 27). Moreover, several reports have shown that the activation of phosphatidylinositol 3-kinase (PI3K)/Akt can phosphorylate GSK3β at Ser9, which leads to the inactivation of GSK3β, and then inhibit the degradation of β-catenin (28–31). And the accumulation and nuclear translocation of β-catenin sustain the transcription of stemness genes including OCT4 (17). Whether these pathways are indeed regulated by FSH in granulosa cells are not known.

In this study, we investigated the cellular and molecular mechanisms by which gonadotropin regulate OCT4 expression during the preantral follicle growth. We demonstrated that FSH regulates OCT4 expression, which is related to the GSK3β/β-catenin pathway. And these responses appeared to be mediated by the PI3K/Akt pathway.

## Materials and Methods

### Reagents and Antibodies

All reagents and chemicals used in present study were purchased from Sigma-Aldrich (St. Louis, MO, USA), unless otherwise indicated. M199 was purchased from Gibco Bethesda Research Laboratories (Grand Island, NY, USA). PI3K inhibitor (LY294002) and Akt inhibitor (API-2) were purchased from Selleck (Selleck Chemicals, Houston, TX, USA). The Cell Counting Kit-8 (CCK-8) was used to analyze cell viability, which was purchased from Dojindo (Dojindo, Kumamoto, Japan). Lipofectamine 3000 was purchased from Invitrogen (Invitrogen, Carlsbad, CA). Rabbit polyclonal anti-OCT4 (ab19857), rabbit monoclonal anti-β-catenin antibody (ab32572), rabbit monoclonal anti-GSK3β antibody (ab3291), rabbit monoclonal anti-Phospho-GSK3β(Ser9) antibody (D3A4), and rabbit polyclonal anti-GAPDH (ab9485) were purchased from Abcam (Abcam, USA). Rabbit monoclonal anti-Phospho-GSK3β (Ser9) antibody (D3A4), rabbit polyclonal anti-phospho-Akt antibody (#9271) and rabbit polyclonal anti-Akt antibody (#9272) were from Cell Signaling Technology (Cell Signaling, USA). Horse radish peroxidase (HRP)-conjugated anti-rabbit IgG was from Santa Cruz Biotechnology, Inc. (Santa Cruz, Beijing). Revert Aid First Strand cDNA Synthesis Kit, TRIzol Reagent were obtained from Thermo Fisher Scientific Inc. (Thermo Scientific, USA), SYBR Green PCR kit was purchased from Bio-Rad (Richmond, CA, USA). PCR primers for OCT4 and 18S rRNA were from Sunbiotech Inc. (Beijing Sunbiotech Co., Ltd. China).

### Animal Experiments

Kunming White female mice (outbreed strain, 21-day old) were purchased from the Beijing Vital Laboratory Animal Technology Co. (Beijing, China). Mice were maintained under constant conditions of temperature (24–26°C) and humidity (60 ± 2%) with a 12/12-h light/dark cycle and received pathogen-free water and food for maintenance. All animal treatment procedures were in accordance with the Principles of the Care and Use of Laboratory Animals and China Council on Animal Care and were approved by the Institutional Animal Care and Use Committee of Capital Normal University. Mice were injected subcutaneously (s.c.) with 10 international units (IU) of eCG in 100 μL of phosphate buffered saline (PBS) containing 0.2% (w/v) bovine serum albumin (BSA) or PBS alone. In some experiment, mice were injected subcutaneously with DES (1 mg/day; 3 days), and ovaries were collected at 72 h after euthanized by cervical dislocation.

### Primary Culture of Granulosa Cells

Granulosa cells were collected by follicular puncture with a 26.5-gauge needle. And cell number and viability were estimated by Trypan blue dye-exclusion test. Granulosa cells (9 ×10^5^ per well in six well plate) were plated with 2 ml of M199 medium [supplemented with HEPES (10 mM), streptomycin (100 μg/ml), penicillin (100 U/ml), and fungizone (0.625 μg/ml)] containing fetal bovine serum (10%, wt/vol) under a humidified atmosphere of 95% air and 5% CO_2_. The cells were treated with FSH (100 ng/ml) for different duration. In some experiments, cells were treated with OCT4 activator O4I2 (12.5 μM) for 24 h or pretreated with PI3K inhibitor LY294002 (10 μM), Akt inhibitor API-2 (10 μM) 1 h or GSK3β inhibitor CT99021 (15 μM) 12 h before hormone treatment, respectively.

### RNA Interference

The siRNA was transfected into granulosa cells according to the instruction of the manufacturer's protocol. Granulosa cells were transfected (48 h) with OCT4 or β-catenin siRNA (GenePharma) and scrambled sequence control (GenePharma), using Lipofectamine 3000 (Invitrogen) according to manufacturer's instructions.

### Protein Extraction and Western Blotting

Western blotting analysis was performed as described previously (32, 33). Briefly, the homogenate was centrifuged (14,000 × g, 4°C, 30 min) for collecting the supernatant after ovaries homogenization. For granulosa cells, whole-cell lysates were prepared by incubating cell pellets in lysis buffer. Meanwhile, Nuclear and Cytoplasmic Protein Extraction Kit (Beyotime P0027) was used to collect the nuclear and cytoplasmic protein. Thirty microgram (depending on individual experiments) of cell lysates were subjected to SDS-PAGE with 4.5% stacking and 10% separating gels. Proteins were electrophoretically transferred to nitrocellulose membrane. And then, the membrane were incubated (4°C, overnight) with diluted primary antibody [polyclonal anti-OCT4 (1:1,000), polyclonal anti-GSK3β (1:5,000), polyclonal anti-phospho-GSK3β (1:1,000), polyclonal anti-β-catenin (1:5,000), polyclonal anti-Akt (1:1,000), polyclonal anti-phospho-Akt (Ser473) (1:1,000) or GAPDH (1:10,000)], respectively, followed by HRP-conjugated secondary antibody (1:1,000–1:10,000; 1.5 h, RT). Peroxidase activity was visualized with the ECL kit according to the manufacturer's instructions. Protein content was determined by densitometrically scanning the exposed x-ray film. Immunoreactions signals were analyzed using gel-pro Analyzer 4.0.

### Real-Time Quantitative PCR Analysis

The cDNA was synthesized form 0.2 μg total RNAs which were extracted with TRIzol Reagent (Invitrogen, Carlsbad, CA). And the mRNA abundance of target genes was analyzed by real-time PCR and normalized to 18S rRNA. Specific primer pairs used in the experiments are listed in Table 1. Data were analyzed by the 2^−ΔΔCT^ method (34).

**Table 1:** Primer sequence used for real-time quantitative PCR.

**Target gene**	**GenBank accession No**.	**Primer sequence**	**Product Size, bp**	**Annealing temperature, ^**°**^C**
OCT4	NM_013633	F:5′ -GAGGAGTCCCAGGACATGAA−3′ R:5′-AGATGGTGGTCTGGCTGAAC−3′	122	62
18S rRNA	NM_008084.3	F:5′- TGGCCTTCCGTGTTCCTAC−3′ R:5′- GAGTTGCTGTTGAAGTCGCA−3′	178	60

### Analysis of Cell Viability

The cell viability was measured by Cell Counting Kit-8 (Dojindo, Kumamoto, Japan) (35). Briefly, CCK-8 solution (10 μL) was added to each well after treatment, and the cells were incubated for an additional 2 h at 37°C. The OD values were recorded using a microplate reader at 450 nm. And the mean OD values for each treatment were used as the index of cell viability.

### Immunofluorescence Cell Staining

Granulosa cells were cultured in poly-D-lysine (0.05% wt/vol; Sigma) coated 8-well glass culture slides (Becton, Dickinson and Co) for immunofluorescence analysis. After treatment, cells were fixed in 4% paraformaldehyde in PBS for 30 min, permeabilized with 0.1% Triton X-100 in PBS for 5 min, and then blocked with 5% BSA in PBS for 30 min. And then, cells were incubated with monoclonal anti-β-catenin (1:200 dilution in blocking solution) at 4°C for overnight. After washing three times with PBS, cells were then incubated with Alexa Fluor 488-conjugated secondary antibody (1:100 dilution in blocking solution; Jackson ImmunoResearch, Lancaster, PA) for 1.5 h at room temperature. After incubating with DAPI, the coverslips were mounted on object slides using fluorescentmounting medium. Immunofluorescence was visualized using an immunofluorescence microscope (Olympus BX51), and images were recorded by using Laser Scanning Microscope LSM 780 (ZEISS, Jena, Germany).

### Statistical Analysis

All the results are expressed as means ± SEM of at least three independent experiments. The statistical differences between treatments were calculated with unpaired or *t*-test, one-way or two-way (repeated-measure) ANOVA (Prism 5.0 statistical software; GraphPad Software, Inc., San Diego, CA). When significant differences were found, means were compared by the Bonferroni post-test. *P* < 0.05 were considered to be statistically significant.

## Results

### Effects of eCG on GSK3β, β-Catenin, and OCT4 Expression

**Figure 1:**
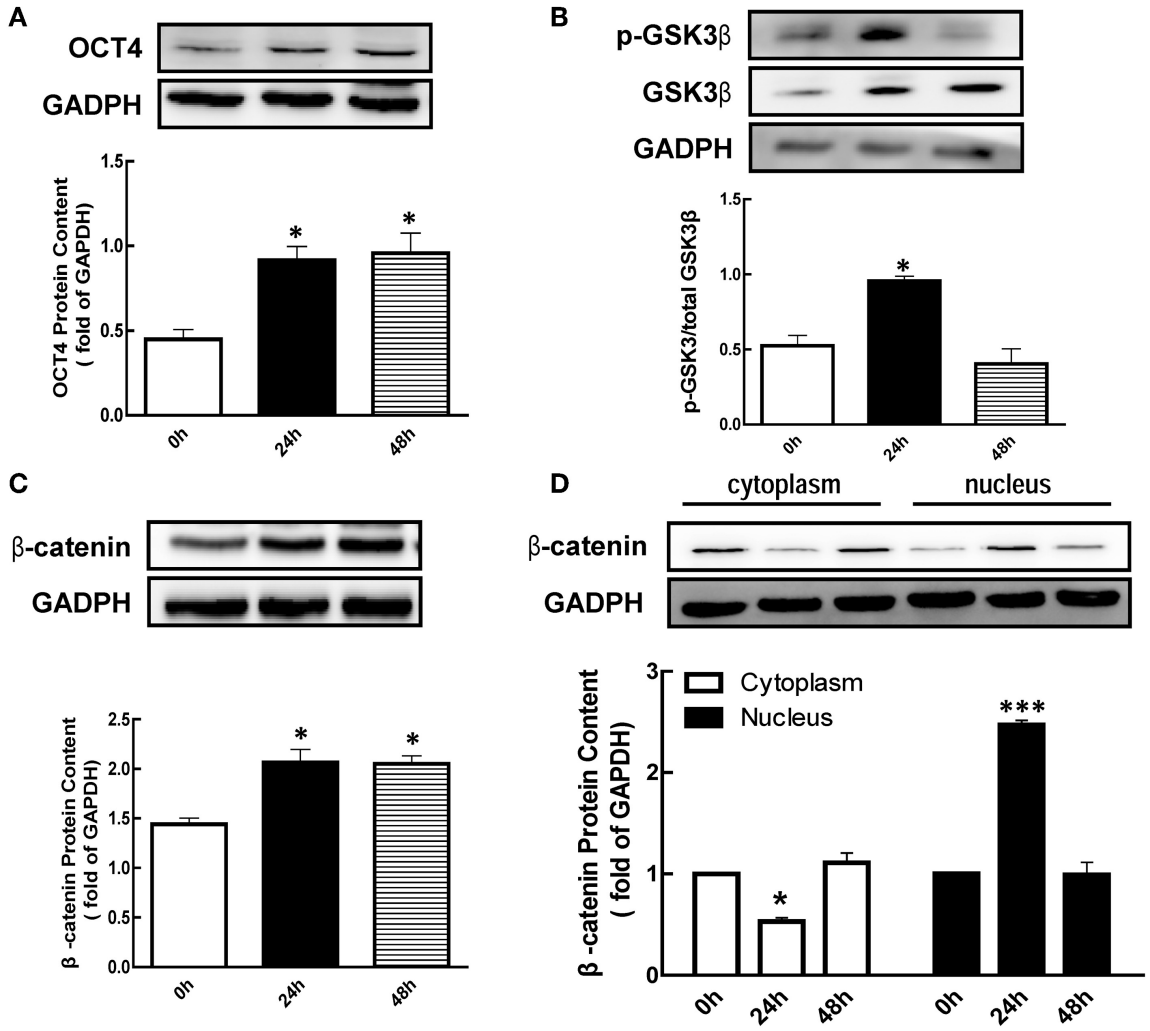
Effect of eCG on OCT4, β-catenin, and GSK3β expression in the ovary. Immature mice were injected with 10 international units (IU) equine chorionic gonadotropin (eCG) for 0, 24, and 48 h, and then ovaries were collected. **(A–C)** OCT4, GSK3β, and β-catenin expression were assessed by western blotting analysis, respectively. **(D)** β-catenin in the cytoplasm and nucleus were collected and also detected by western blotting analysis. Data are presented as mean ± SEM of three independent experiments. *P < 0.05, ***P < 0.001 compared with 0 h, respectively.

To investigate the effect of eCG on the expression of p-GSK3β, β-catenin, and OCT4 protein in mice ovary, eCG were injected for 24 or 48 h, respectively, and determined ovarian OCT4 content using western blotting analysis. As shown in Figure 1A, the expression of OCT4 was significantly increased 2.04 and 2.13-fold at 24 and 48 h after eCG treatment (*p* < 0.05), respectively.

Meanwhile, we also detected the p-GSK3β (Ser9) level after eCG treatment. The results showed that the level of p-GSK3β (Ser9) was significantly increased 1.82-fold at 24 h (Figure 1B, *p* < 0.05) after eCG treatment, while there is no significant difference at 48 h (Figure 1B). As shown in Figure 1C, the level of β-catenin was significantly increased 1.43 and 1.42-fold at 24 and 48 h after eCG treatment (*p* < 0.05), respectively. In addition, eCG significantly increased β-catenin translocation from the cytoplasmic to the nuclear at 24 h (Figure 1D, *p* < 0.001).

### Effects of OCT4 on FSH-Induced Granulosa Cell Development

**Figure 2:**
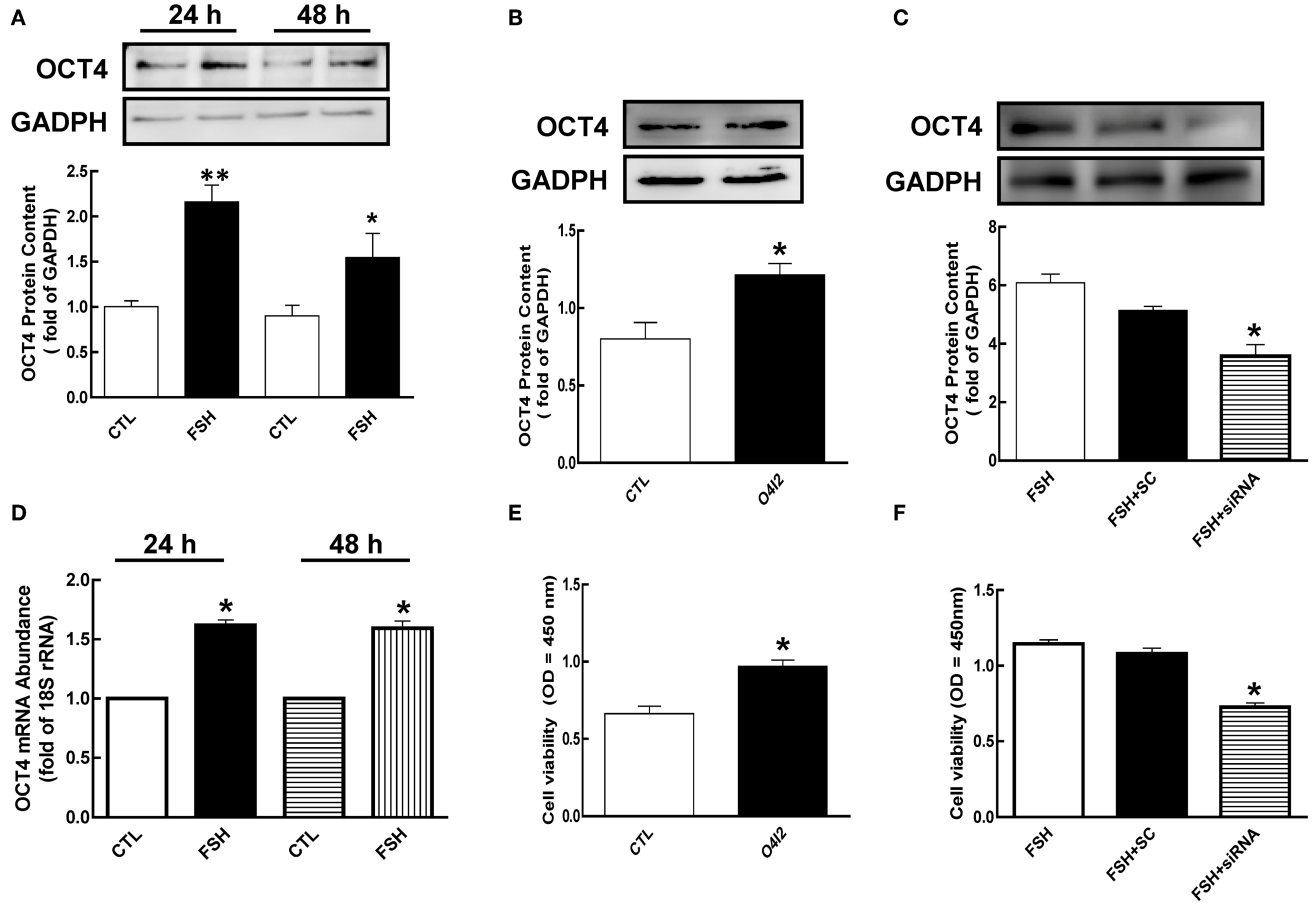
Role of OCT4 in FSH-induced cellular development. **(A)** Granulosa cells were cultured with FSH for 24 and 48 h, and OCT4 expression was assessed by western blotting analysis. **(B)** Granulosa cells were treated with O4I2 (OCT4 activator) for 24 h, and OCT4 protein was assessed by western blotting analysis. **(C)** Granulosa cells were transfected with OCT4 siRNA (scrambled sequence as control, SC) for 48 h using Lipofectamine 3000, and then treated with FSH for another 24 h and detected OCT4 protein. **(D)** Cells were harvested at 24 h for mRNA analysis by real-time PCR. mRNA abundance was normalized using 18S rRNA. **(E)** Granulosa cells were cultured with O4I2 for 24 h, and cell viability was analyzed by CCK-8 assay. **(F)** Granulosa cells were treated with OCT4 siRNA as previously described, and cell viability was also analyzed by CCK-8 assay. Data are presented as mean ± SEM of three independent experiments. *P < 0.05; **P < 0.01 compared with control (CTL, **A,B,D,E**) or FSH alone **(C,F)**, respectively.

To determine whether FSH regulates the expression of OCT4, we cultured granulosa cells with FSH for 24 or 48 h. As shown in Figure 2A, FSH significantly increased OCT4 expression [2.15 (24 h) and 1.72-fold (48 h), *p* < 0.05]. To better understand whether OCT4 content is regulated at the mRNA level by hormone, we determined OCT4 mRNA abundance using real-time PCR analysis. Granulosa cells cultured with FSH for 24 h had higher mRNA levels compared to those cultured without FSH (Figure 2D, *p* < 0.05). Moreover, the expression of OCT4 mRNA was also significantly changed after FSH treatment for 48 h. These results suggest that the regulation of OCT4 expression in granulosa cells by FSH occurs at the transcription level or via translational processing.

To investigate the function of OCT4 in granulosa cell development, we cultured cells with OCT4 activator (O4I2) or siRNA with and without FSH. As shown in Figures 2B,C, OCT4 protein content was significantly increased by O4I2 (*p* < 0.05). Meanwhile, O4I2 significantly induced cellular viability compared that with the control group (Figure 2E, *p* < 0.05). In contrast, cellular viability was significantly attenuated by OCT4 knockdown (Figure 2F, *p* < 0.05).

### β-Catenin Mediated FSH Induced OCT4 Expression

**Figure 3:**
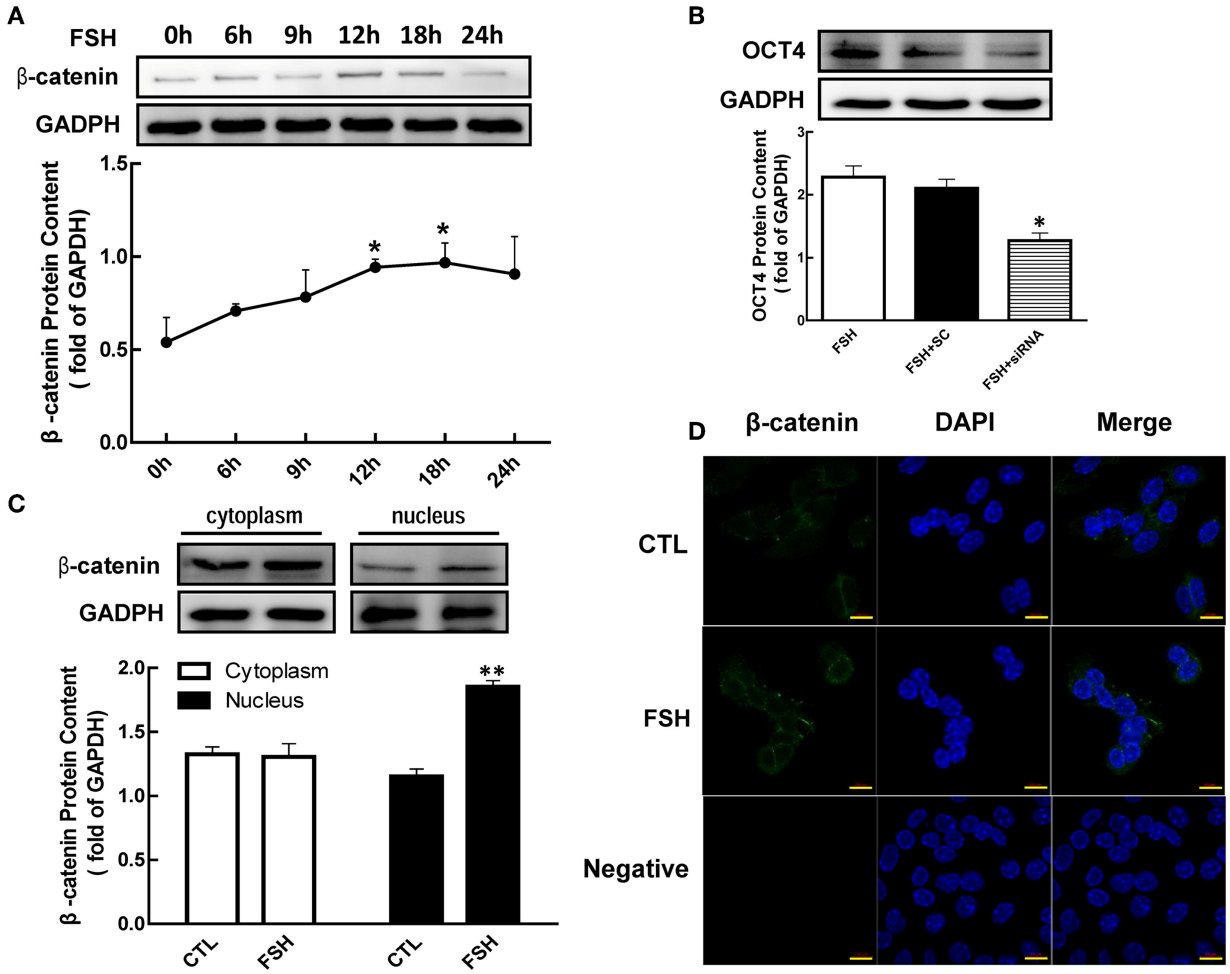
Role of β-catenin in FSH-induced OCT4 expression. **(A)** Granulosa cells were cultured with FSH for different duration, and β-catenin expression was assessed by western blotting analysis. **(B)** Granulosa cells were transfected with β-catenin siRNA (scrambled sequence as control, SC) for 48 h using Lipofectamine 3000, and then treated with FSH for another 24 h. OCT4 protein was assessed by western blotting analysis. **(C)** Granulosa cells were cultured with FSH for 18 h, and β-catenin expression in the cytoplasm and nucleus were assessed. **(D)** FSH induced the shift of β-catenin distribution to the nucleus, which was detected by immunofluorescence. Data are presented as mean ± SEM of three independent experiments. *P < 0.05; **P < 0.01 compared with 0 h **(A)**, FSH alone **(B)**, or control (CTL, **C**), respectively. Bar, 10 μm.

It has been reported that β-catenin is a bifunctional protein as an intracellular signal transduction. To determine whether β-catenin is regulated by hormone, granulosa cells were co-cultured with FSH for 24 h. We noticed that β-catenin protein levels were significantly enhanced by FSH from 1.75 (12 h) to 1.80 (12 h)-fold (Figure 3A, *p* < 0.05). Moreover, FSH also significantly increased β-catenin translocation from the plasma to nucleus (Figure 3C, *p* < 0.01). These results were also confirmed by immunofluorescence cell staining (Figure 3D).

To further determine whether β-catenin is essential for FSH-induced OCT4 expression, we applied siRNA to knockdown β-catenin expression. While OCT4 protein content was up-regulated by the presence of FSH, this effect was significantly attenuated 1.79-fold by β-catenin knockdown (Figure 3B, *p* < 0.05).

### Role of GSK3β in FSH Induced β-Catenin and OCT4 Expression

**Figure 4:**
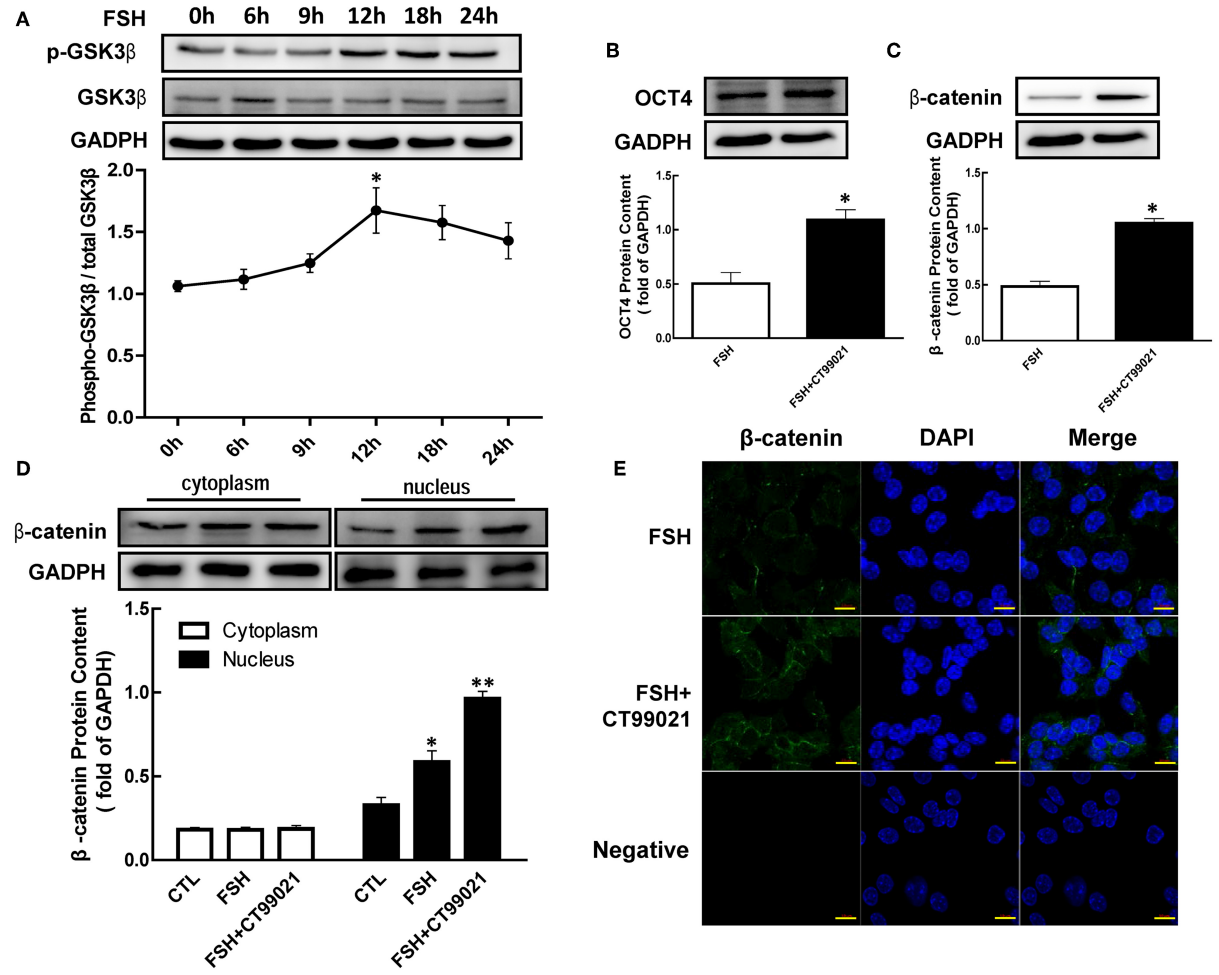
Role of GSK3β on OCT4 and β-catenin regulated by FSH. **(A)** Granulosa cells were cultured with FSH for different duration, and GSK3β expression was assessed by western blotting analysis. **(B,C)** After pretreated with CT99021 (GSK3β inhibitor) for 12 h, granulosa cells were co-cultured with FSH for 18 or 24 h, and then the expression of β-catenin or OCT4 were detected by western blotting analysis, respectively. **(D)** Granulosa cells were cultured with CT99021 for 12 h, and then treated with FSH for 18 h, the β-catenin expression in the cytoplasm and nucleus were assessed by western blotting analysis. **(E)** The expression of β-catenin in the cytoplasm and nucleus were detected by immunofluorescence. Data are presented as mean ± SEM of three independent experiments. *P < 0.05; **P < 0.01 compared with 0 h **(A)**, FSH alone **(B,C)** or control (CTL, **D**), respectively. Bar, 10 μm.

To verify the hypothesis that regulations of β-catenin and OCT4 by hormone were mediated by phosphorylating GSK3β (Ser9), we cultured granulosa cells with FSH, and the content of p-GSK3β were detected by western blotting. As shown in Figure 4, the expression of p-GSK3β (Ser9) was increased with the duration of treatment, and reaching a peak at 12 h after FSH treatment with a maximum increase of 1.58-fold (Figure 4A, *p* < 0.05).

To determine the role of GSK3β in hormone-induced β-catenin and OCT4 expression, granulosa cells were pretreated with CT99021 (GSK3β activity inhibitor) for 12 h, and then treated with FSH for another 18 or 24 h. The results showed that the expression of β-catenin (Figure 4C, *p* < 0.05) and OCT4 (Figure 4B, *p* < 0.05) induced by FSH were significantly enhanced by CT99021. Moreover, CT99021 also increased FSH-induced translocation of β-catenin by 1.65-fold, which was also confirmed by immunofluorescence results (Figures 4D,E, *p* < 0.05; *p* < 0.01).

### Involvement of the PI3K/Akt Pathway in FSH-Induced Genes Expression

**Figure 5:**
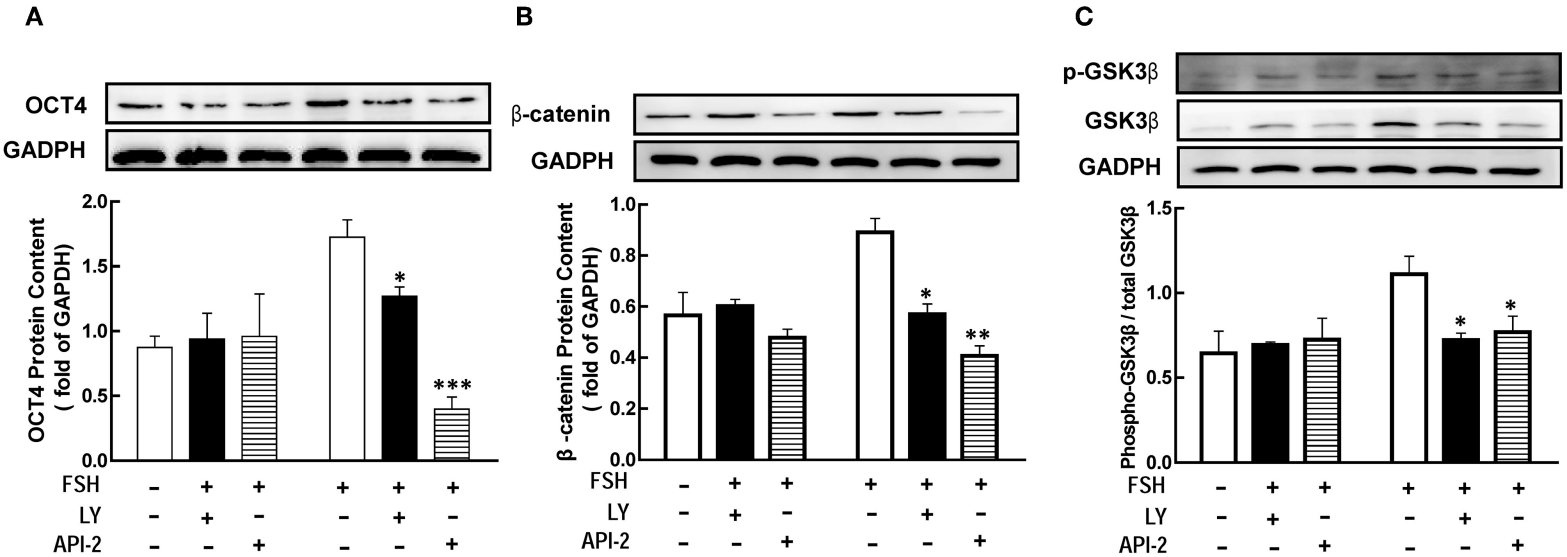
The PI3K/Akt pathway mediated FSH-induced genes expression. Granulosa cells were cocultured with inhibitors LY294002 (LY, 10 μM) or API-2 (10 μM) for 1 h before hormone treatment. The expression of OCT4 **(A)**, β-catenin (B) and GSK3β **(C)** were assessed by western blotting analysis after FSH treatment for 24, 18, or 12 h, respectively. Data are presented as mean ± SEM of three independent experiments. *P < 0.05; **P < 0.01, ***P < 0.001 compared with FSH.

Previous studied had showed that FSH promoted granulosa cells development through PI3K/Akt pathway (8, 9). To determine whether PI3K/Akt signaling is necessary for FSH-mediated OCT4 expression, we applied LY294002 or API-2 1 h before FSH treatment. The results showed that the level of OCT4 was significantly lower than in the group untreated with inhibitors (Figure 5A, *p* < 0.05; *p* < 0.01). The expression of β-catenin or GSK3β after hormones treatment for 18 or 12 h were also detected, respectively. The same effects were also showed on β-catenin and p-GSK3β expression (Figures 5B,C, *p* < 0.05).

## Discussion

In the present study, we have demonstrated that FSH-induced granulosa cells development through up-regulating OCT4 expression in mice. These responses are mediated by p-GSK3β (Ser9), β-catenin expression, and translocation. Moreover, the activated PI3K/Akt pathway is also involved in these regulations. To the best of our knowledge, it is the first study to prove that OCT4 plays a crucial role in FSH-induced development in granulosa cells at the preantral to early antral transition stage of follicles.

It is well-known that gonadotropin is important for follicle development, which regulates OCT4 expression and functions in ovary (19). As a transcription factor, OCT4 is involved in the regulation of many genes. In the present study, the expression of ovarian OCT4 was increased after treatment, which is consistent with the previous study (20). Meanwhile, the expression of OCT4 in granulosa cells was also significantly increased by FSH. Knockdown of OCT4 expression by siRNA decreased FSH-induced cell growth, which indicates OCT4 is important to the granulosa cell development. Moreover, FSH increases ovarian cells growth by regulating OCT4 expression, which is possibly mediated by FSHR (Follicle stimulating hormone receptor). It has been reported that both FSHR and OCT4 are expressed in the oocyte of primordial follicle. And the co-localization of FSHR and OCT4 are changed along with follicular growth, which are decreased in oocyte and increased in peripheral granulosa cells in growing follicles (36).

The previous reports show that β-catenin is very important to the stem cell proliferation (22) and hair follicle regeneration (21) by regulating OCT4 expression. It is possible that FSH-induced OCT4 expression is mediated by β-catenin since β-catenin is negatively related to follicular atresia (37). The present study showed that β-catenin was significantly increased in ovary, and then was transported from the cytoplasm to the nucleus after eCG treatment. The patterns are similar with that eCG-induced OCT4 expression *in vivo*. The results in granulosa cells *in vitro* confirm the hypothesis that FSH increased β-catenin expression and translocation to nuclear, which are essential to OCT4 expression. Moreover, the regulations on follicular development by β-catenin may be also mediated by regulating estrogen biosynthesis since β-catenin is a transcriptional regulator of CYP19A1 (38).

It is also well-known that β-catenin is degraded after forming a complex with the activated GSK-3β (39). GSK3β, as a serine/threonine kinase of the Wnt/β-catenin pathway, is a negative regulator of Wnt signaling, which phosphorylates β-catenin residues, thereby degrading β-catenin via proteasome (40). However, once GSK-3β is phosphorylated to an inactive form (41) (Ser9), β-catenin is transferred to the nucleus without degradation and start to transcript (43, 44). After interaction with TCF3 (Transcription factor 3), nuclear β-catenin induce the expression of the plasticity factors including OCT4, which in turn promotes hair follicle regeneration (21). Our results showed that the expression of p-GSK3β (Ser9) in ovary is significantly increased after eCG treatment *in vivo*, which was accompanied by the increased β-catenin expression. Moreover, FSH increased p-GSK3β level and β-catenin expression in granulosa cells *in vitro*. It is possible that the increased intracellular β-catenin-induced by gonadotropin is due to the phosphorylation of GSK3β at Ser9 as the inactive form in the present study. Moreover, gonadotropin also increased the translocation of β-catenin from the cytoplasm to the nucleus. In addition, FSH-induced OCT4 expression was attenuated by LY294002 and API-2, respectively. The results indicate that the regulation of hormone on OCT4 is mediated by the PI3K/Akt pathway. It is consistent with the previous reports that the activated Akt inactivates GSK3β and inhibits β-catenin degradation by phosphorylating GSK3β at Ser9 (28–31, 42).

**Figure 6:**
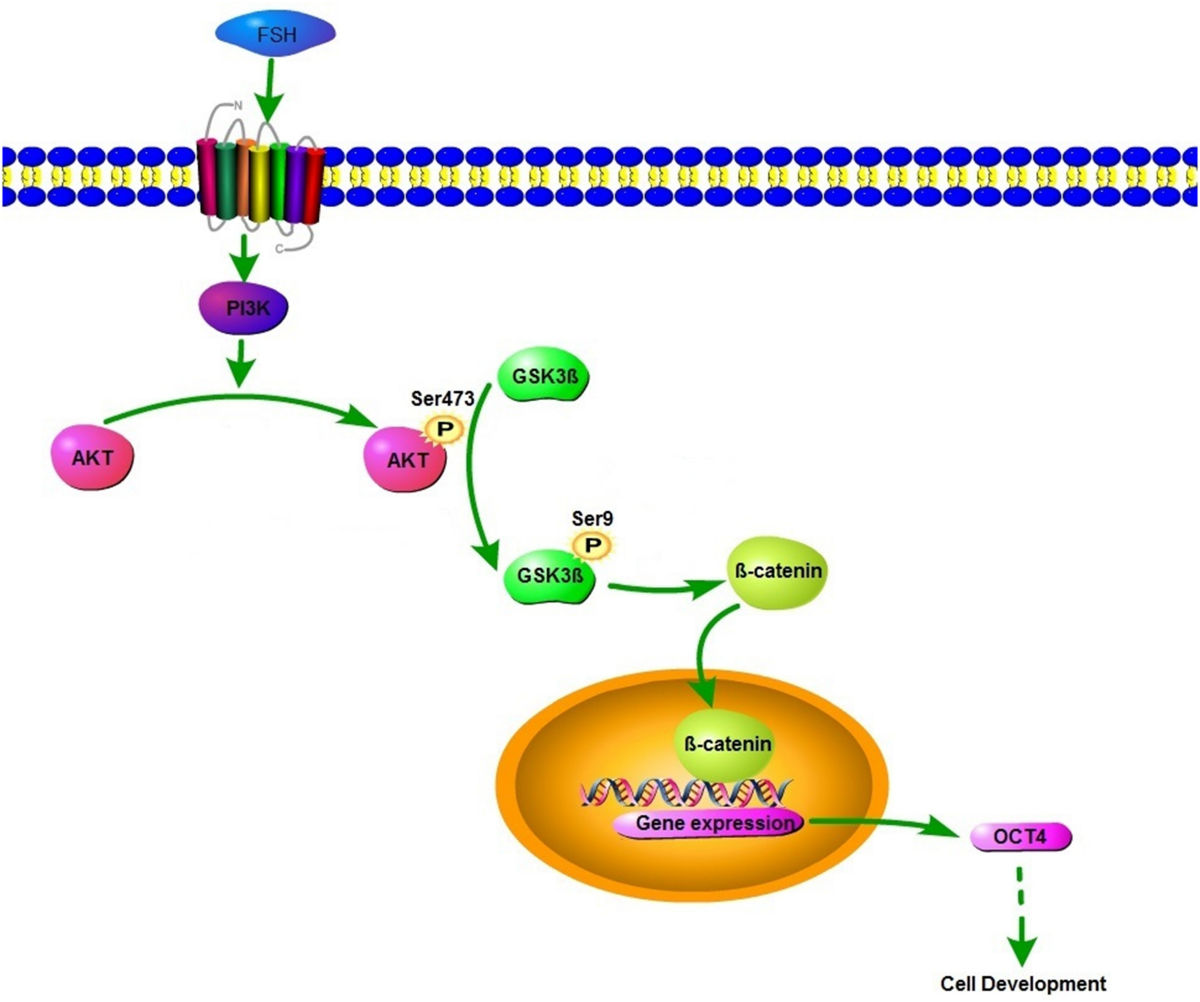
Schematic diagram of the roles of FSH on OCT4 expression.

In conclusion, our findings demonstrate that OCT4 is a novel positive regulator for the granulosa cell development in the early stage of follicles. As indicated by the model (Figure 6), FSH induces the expression of OCT4 in granulosa cells through GSK3β/β-catenin pathway and promotes cellular development, and these regulatory processes are all mediated by the PI3K/Akt signaling pathway. The results enhance our understanding of the role of OCT4 in ovarian cells.

## Data Availability Statement

All datasets generated for this study are included in the article/supplementary material.

## Ethics Statement

The animal study was reviewed and approved by Institutional Animal Care and Use Committee of Capital Normal University.

## Author Contributions

DH and QW performed the experiments and analyzed the data. CZ, XM, and DH interpreted the results of the experiments. XW and KX prepared the figures. YT, WL, KX, and XH contributed to the discussion. CZ designed the research and wrote the manuscript.

### Conflict of Interest

The authors declare that the research was conducted in the absence of any commercial or financial relationships that could be construed as a potential conflict of interest.

## Abbreviations

FSH: follicle-stimulating hormone
LH: luteinizing hormone
GnRH: gonadotropin-releasing hormone
E_2_: estradiol
PI3K: phosphoinositide 3-kinase
OCT4: octamer-binding transcription factor 4
GSK3β: glycogen synthase kinase 3-beta gene
FSHR: follicle stimulating hormone receptor
TCF3: transcription factor 3
HPG: hypothalamus–pituitary–gonadal axis.
